# Sex differences following percutaneous coronary intervention or coronary artery bypass surgery for acute myocardial infarction

**DOI:** 10.1186/s13293-022-00427-1

**Published:** 2022-04-27

**Authors:** Donna Shu-Han Lin, Yu-Sheng Lin, Jen-Kuang Lee, Hsien-Li Kao

**Affiliations:** 1 Division of Cardiology, Department of Internal Medicine, National Taiwan University Hospital, Hsin-Chu Branch, Hsinchu, Taiwan; 2 Division of Cardiology, Department of Internal Medicine, National Taiwan University Hospital, No. 7, Chung-Shan South Road, Taipei, 100 Taiwan; 3 Division of Cardiology, Department of Internal Medicine, Chang Gung Memorial Hospital, Chiayi, Taiwan; 4 College of Medicine, Graduate Institute of Clinical Medical Sciences, Chang Gung University, Taoyuan City, Taiwan; 5 Department of Internal Medicine, National Taiwan University College of Medicine, Taipei, Taiwan; 6 Department of Laboratory Medicine, National Taiwan University College of Medicine, Taipei, Taiwan; 7 Cardiovascular Center, National Taiwan University Hospital, Taipei, Taiwan; 8 Telehealth Center, National Taiwan University Hospital, Taipei, Taiwan

**Keywords:** Acute myocardial infarction, Percutaneous coronary intervention, Coronary artery bypass graft surgery, Sex, Heart failure

## Abstract

**Background:**

Women have been underrepresented in the literature; the effects of female sex on outcomes in patients with acute myocardial infarction (AMI) remain unclear.

**Objectives:**

This study compares the real-world outcomes of women and men with AMI who have undergone revascularization via percutaneous coronary intervention (PCI) or coronary artery bypass graft surgery (CABG).

**Methods:**

This is a retrospective cohort study utilizing data from the Taiwan National Health Insurance database. We identified patients who were admitted for AMI and who underwent coronary revascularization during the index admission period between January 1, 2001, and December 31, 2013. Patients were then categorized based on the treatment received into PCI and CABG groups. In-hospital and long-term outcomes were compared between women and men in each group. Interaction tests were then performed to determine whether the differences between sexes were modified by the mode of revascularization. Analyses were repeated after propensity score matching between women and men in each group to minimize possible confounders. We also conducted subgroup analyses, stratifying by the presence of diabetes mellitus, congestive heart failure, and chronic kidney disease.

**Results:**

We enrolled 67,534 patients who met the inclusion criteria in the analysis; 60,207 patients had undergone PCI (13,514 female and 46,693 male), while 7327 patients had received CABG (1762 female and 5565 male). Prior to matching, enrolled female patients were older on average, with more comorbidities. In-hospital and long-term outcomes were worse in women, particularly in the PCI group. After matching, the incidence of hospitalization for heart failure (HHF) was higher in women (10.4% vs 8.0%, OR 1.32, 95% CI 1.22–1.43), with fewer repeat revascularizations (28.1% vs 32.4%, OR 0.84, 95% CI 0.81–0.88). Both observations were more pronounced in the PCI group (HHF: *P* for interaction = 0.0496; repeat revascularization: *P* for interaction = 0.021).

**Conclusions:**

Women presenting with AMI exhibited worse in-hospital and long-term outcomes than men, especially among women who received PCI as the initial mode of revascularization. Women who underwent PCI were more likely to be admitted for heart failure during follow-up. Possible socioeconomic inequalities or a distinct pathobiology of cardiac ischemia between sexes may underlie these results; thus, further investigation is needed.

**Supplementary Information:**

The online version contains supplementary material available at 10.1186/s13293-022-00427-1.

## Highlights

- Among patients with acute myocardial infarction (AMI), women were older, had more comorbid conditions, and were less likely to be discharged with optimal medical therapy than their male counterparts.
- In-hospital and long-term outcomes were worse among women compared to men, particularly in those who received percutaneous coronary intervention (PCI) as the mode of revascularization for AMI. Despite worse in-hospital survival, women were less likely to receive mechanical cardiac support.
- After propensity score matching between women and men for baseline characteristics, the incidence of hospitalization for heart failure was higher among women during long-term follow-up, especially among patients who had undergone PCI. Contrarily, the incidence of repeat revascularization procedures was lower in women in the long term.
- Mechanisms underlying cardiac ischemia likely differ between women and men, and socioeconomic inequalities that influence treatment of female patients are also possible.

**Supplementary Information:**

The online version contains supplementary material available at 10.1186/s13293-022-00427-1.

## Introduction

Cardiovascular diseases (CVDs) are the leading causes of mortality and morbidity worldwide. According to WHO statistics, CVDs were responsible for approximately 17.9 million deaths globally in 2016. Despite the conventionally reported protective effects of female sex, 35% of total deaths in women were attributable to CVD in 2019, with ischemic heart disease being the primary cause [1]. The traditional risk factors of coronary artery disease (CAD)—namely, hypertension, dyslipidemia, diabetes, obesity, and smoking—have become more prevalent among modern women. Furthermore, women are at risk for several sex-specific conditions such as gestational hypertension or diabetes, premature menopause, contraceptive use, and polycystic ovary syndrome, which are related to CVD.

Despite its significance, CAD in women continues to be underacknowledged due to social, economic, and cultural factors, contributing to underdiagnosis and undertreatment of CAD in women [1]. Likewise, women are often underrepresented in clinical trials. The participation-to-prevalence ratios of women in clinical trials on CAD, acute coronary syndrome (ACS), and heart failure have reportedly ranged from 0.48 to 0.67 [2]. For example, of the 1800 patients with complex CAD enrolled in the SYNTAXES [3] study, only 22.3% were female. Similar proportions of female patients (23.1%) were seen in the EXCEL [4] study, which compared the efficacy and safety of percutaneous coronary intervention (PCI) with a drug-eluting stent (DES) against coronary artery bypass grafting surgery (CABG) for unprotected left main disease. In several recently published studies investigating antiplatelet use after PCI, females constituted only 23.3–30.3% of the enrolled participants [5–7]. The efficacy of many treatments is thus insufficiently investigated in female patients.

Outcomes among female patients were compared to those among men in the post hoc analyses of the aforementioned trials, each with distinct conclusions. Although women had consistently worse outcomes in general, the results from each study were variable after adjustment for age and comorbidities. Similarly, revascularization for CAD and AMI by PCI or CABG have been compared between women and men in several real-world cohort studies, also with conflicting results. Furthermore, there is a paucity of data regarding how sex affects the outcomes of CAD across various levels of disease severity and different modes of revascularization. Therefore, additional evidence is needed to clarify sex differences in the clinical outcomes of CAD patients.

This study compares the real-world outcomes of women and men with AMI who underwent revascularization by PCI or CABG. Patients were enrolled through a nationwide database that includes nearly 100% of the population in Taiwan. Outcomes were assessed before and after propensity score matching to account for the effects of sex in general and for sex-specific physiological differences. Interaction tests were utilized to evaluate whether the effects of sex differed among patients who received different types of revascularization.

## Methods

### Data source

The Taiwan National Health Insurance (NHI) program is a single-payer system established in March 1995. Registration for the NHI program has been compulsory in Taiwan since March 1995; it currently covers medical expenditures for over 99.8% of Taiwan’s population, which stands at 23.8 million people. The NHI provides affordable, high-quality healthcare that meets global standards. The National Health Insurance Research Database (NHIRD) utilized in this study is maintained by the National Health Research Institutes. As patient data were de-identified upon collection, this study was waived from informed consent and full review by the Ethics Institutional Review Board of Taiwan University Hospital. Details regarding the NHI program and the NHIRD have been reported in previous publications [8–10].

### Study cohort

Patients who were admitted for AMI and who had undergone coronary revascularization (PCI or CABG) during the index admission period between January 1, 2001, and December 31, 2013, were identified using the International Classification of Diseases, Ninth Revision, Clinical Modification (ICD-9-CM) codes of 410.x [11]. The first admission for AMI was assigned as the index admission if the patient suffered from 2 or more admissions for AMI during the study period. PCI was identified via the presence of Taiwan NHI reimbursement codes of 33,076 (one vessel), 33,077 (two vessels), or 33,078 (three vessels) for PCI in a combination with the supply codes for coronary stent. CABG was identified by the presence of Taiwan NHI reimbursement codes of 68,023 for one vessel, 68,024 for two vessels, and 68,025 for three vessels.

**Fig. 1:**
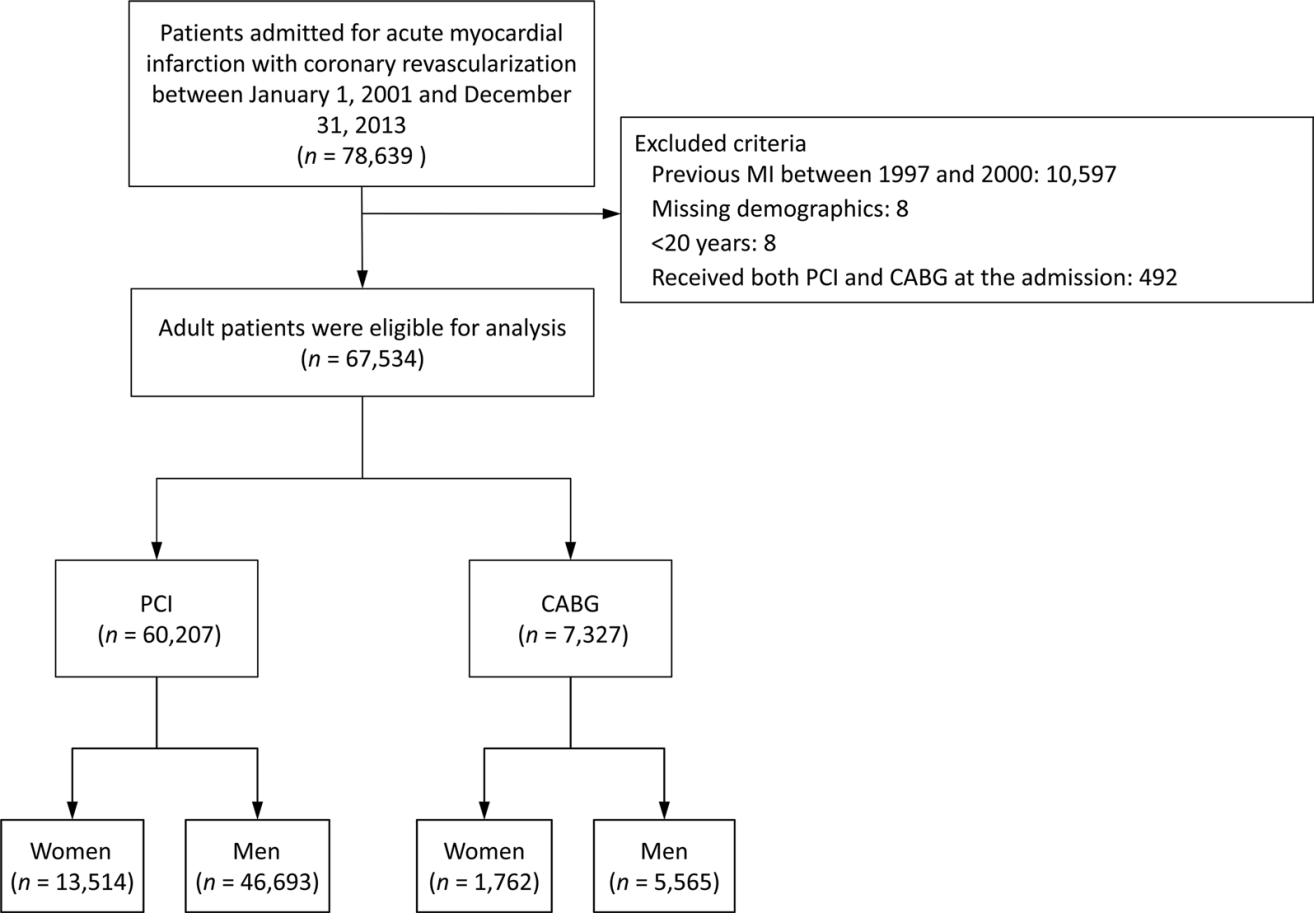
Flowchart for patient selection. *MI* myocardial infarction, *PCI* percutaneous coronary intervention, *CABG* coronary artery bypass grafting

A total of 78,639 patients were found. The exclusion criteria include (1) missing demographic data (*n* = 8); (2) a history of previous myocardial infarction (MI) between 1997 and 2000 (*n* = 10,597); (3) age younger than 20 years (*n* = 8), or (4) receipt of both PCI and CABG during the index admission (*n* = 492). Patients were then categorized into two groups according to the means of coronary revascularization: the PCI group and the CABG group. Each group was then further divided into two subgroups by sex (Fig. 1). Patients were followed until the occurrence of any study outcomes or until December 31, 2013—whichever came first.

### Covariates

The covariates incorporated in this study are as follows: (1) age; (2) comorbid conditions (diabetes mellitus, hypertension, dyslipidemia, a history of hospitalization for heart failure [HHF], peripheral arterial disease [PAD], chronic obstructive pulmonary disease [COPD], liver cirrhosis, cancers, autoimmune diseases, prior hospitalization for stroke, chronic kidney disease [CKD], and end-stage renal disease [ESRD] under dialysis); (3) the Charlson Comorbidity Index (CCI) score; (4) a history of significant bleeding; (5) the type of medical institute, and 6) antiplatelet agent or other medication use at discharge. A history of significant bleeding was defined as hospitalization due to bleeding (designated as “major bleeding”), gastrointestinal bleeding, intracranial hemorrhage (ICH), or anemia. All of these diseases (except heart failure [HF], stroke, and bleeding events) were identified if they were present at least twice in outpatient diagnoses or if they appeared in any inpatient diagnoses over the previous year. A history of HF, stroke, and bleeding events (except anemia) was defined as having any one of these inpatient diagnoses preceding the index admission for AMI, which can be tracked back to 1997.

The antiplatelets of interest are aspirin and P2Y12 receptor inhibitors (clopidogrel or ticlopidine); whether the patient was discharged with a single or dual antiplatelet regimen was also recorded. Other drug use examined includes statins, beta-blockers, angiotensin-converting enzyme inhibitors/angiotensin receptor blockers, antidiabetic agents, anticoagulants, proton pump inhibitors, and non-steroidal anti-inflammatory drugs. Regarding details of coronary revascularization, the number of coronary arteries intervened or grafted was extracted. In patients who had undergone PCI, the type of stents deployed (bare metal stent [BMS] or drug-eluting stent [DES]) and the number of stents used were also captured. For patients who received CABG, other covariates regarding surgical details include the use of cardiopulmonary bypass pumps (on-pump versus off-pump), performance of endarterectomy, concomitant valve surgery, and the type of valvular surgery performed (if available). Most of these covariates were identified using Taiwan NHI reimbursement codes, while some were identified using ICD-9-CM procedural codes, including the type of valvular surgery. All ICD diagnostic, therapeutic, and pharmacological codes of the included covariates are provided in Additional file 1: Table S1.

### Outcomes

The outcomes of interest in this study include in-hospital and long-term outcomes. In-hospital outcomes include in-hospital mortality, the use of intra-aortic balloon pumps (IABPs), the use of extra-corporeal membrane oxygenation (ECMO) support, ischemic stroke, pneumonia, gastrointestinal bleeding, new-onset dialysis, prolonged ventilation, length of hospital stay (LOS), length of stay in the intensive care unit (ICU), and total medical expenditure. Prolonged ventilation was defined as dependence on mechanical ventilation for seven days or longer. IABP and ECMO use were designated as surrogate indicators for disease severity. Information on IABP, ECMO, dialysis, ventilation, and ICU was captured using the Taiwan NHI reimbursement codes. Pneumonia and gastrointestinal bleeding were confirmed using the ICD-9-CM codes of discharge diagnoses. Records on in-hospital death, LOS, and length of stay in the ICU were available from inpatient claims data.

The primary long-term outcomes investigated are cardiovascular death (CV death), AMI, ischemic stroke, and a composite of these outcomes. The secondary long-term outcomes studied are death from any cause, HHF, and repeat coronary revascularization by either PCI or CABG. Long-term safety outcomes include major bleeding, gastrointestinal bleeding, and ICH. CV death has been specified in line with the Standardized Definitions for Cardiovascular and Stroke Endpoint Events in Clinical Trials by the FDA in the United States. The occurrence of AMI, ischemic stroke, heart failure, and bleeding events is defined by subsequent hospitalization for these causes; these occurrences were identified based on the principal discharge diagnosis. Dates and causes of death are available in the Registry for Beneficiaries sub-database of the NHIRD. Revascularization via PCI or CABG was detected through the inpatient claims data, with reimbursement codes for the Taiwan NHI. Most of these diagnostic codes have been validated in previous National Health Insurance Research Database (NHIRD) studies [11–16].

### Statistical analysis

In-hospital outcomes were compared between female and male patients in both the PCI and CABG groups using logistic regression for the binary outcomes, or using linear regression for the continuous outcomes. The risks of fatal outcomes (i.e., in-hospital mortality, CV death, or all-cause mortality) were compared between the two sexes using the Cox proportional hazard model. Other time-to-event outcomes were compared between women and men using the Fine and Gray subdistribution hazard model, with death during the follow-up period being considered a competing risk. The regression coefficients for females relative to males were further compared between the PCI and CABG groups to assess whether the differences between women and men were consistent across different coronary treatments. A formal interaction test was performed and a two-sided *P* value < 0.05 was considered to be statistically significant.

To evaluate the effect of sex on outcomes and to minimize confounding and selection bias, propensity score matching was conducted. The aforementioned analyses of outcomes were performed in the matched cohorts and termed as the primary analysis. The propensity score, defined as the conditional probability of the background covariates listed in Table 1, was calculated using a multivariable logistic regression model, in which the study groups (1: female and 0: male) were regressed on the selected covariates (listed in Table 1). Matching was done using a greedy nearest neighbor algorithm, with a caliper of 0.2 times the standard deviation of the logit of the propensity score. A random matching order and replacement were not allowed. Female and male patients were matched in a 1:1 ratio in either the PCI or CABG treatment groups. The quality of matching was assessed by the absolute value of standardized difference (STD) between the groups after propensity score matching, where a value of less than 0.1 was considered negligible.

**Table 1:** Demographics, clinical and surgical characteristics of the women versus men in the cohort before propensity score matching

Variable	PCI with stent			CABG		
	Women (*n* = 13,514)	Men (*n* = 46,693)	STD	Women (*n* = 1762)	Men (*n* = 5565)	STD
Age (years)	71.2 ± 11.7	61.8 ± 13.4	0.75	69.3 ± 9.9	65.2 ± 11.7	0.38
Comorbid conditions						
Diabetes mellitus	7374 (54.6%)	15,866 (34.0%)	0.42	1212 (68.8%)	2644 (47.5%)	0.44
Hypertension	10,448 (77.3%)	27,716 (59.4%)	0.39	1420 (80.6%)	3734 (67.1%)	0.31
Dyslipidemia	5901 (43.7%)	21,987 (47.1%)	− 0.07	681 (38.6%)	1942 (34.9%)	0.08
Heart failure hospitalization	1647 (12.2%)	2041 (4.4%)	0.29	265 (15.0%)	435 (7.8%)	0.23
Peripheral arterial disease	807 (6.0%)	1412 (3.0%)	0.14	137 (7.8%)	339 (6.1%)	0.07
Prior stroke hospitalization	2550 (18.9%)	4998 (10.7%)	0.23	353 (20.0%)	901 (16.2%)	0.10
COPD	945 (7.0%)	4203 (9.0%)	− 0.07	111 (6.3%)	678 (12.2%)	− 0.20
Liver cirrhosis	245 (1.8%)	905 (1.9%)	− 0.01	19 (1.1%)	122 (2.2%)	− 0.09
Malignancy	773 (5.7%)	1860 (4.0%)	0.08	60 (3.4%)	177 (3.2%)	0.01
Autoimmune disease	434 (3.2%)	552 (1.2%)	0.14	43 (2.4%)	65 (1.2%)	0.10
Chronic kidney disease	3689 (27.3%)	7024 (15.0%)	0.30	598 (33.9%)	1480 (26.6%)	0.16
Dialysis	980 (7.3%)	1371 (2.9%)	0.20	115 (6.5%)	240 (4.3%)	0.10
Charlson’s Comorbidity Index score	3.4 ± 2.1	2.5 ± 1.8	0.47	3.8 ± 2.0	3.1 ± 2.0	0.32
Bleeding history						
Major bleeding	964 (7.1%)	2180 (4.7%)	0.10	94 (5.3%)	247 (4.4%)	0.04
GI bleeding	2758 (20.4%)	5721 (12.3%)	0.22	305 (17.3%)	734 (13.2%)	0.11
ICH	200 (1.5%)	548 (1.2%)	0.03	22 (1.2%)	56 (1.0%)	0.02
Anemia	506 (3.7%)	608 (1.3%)	0.16	49 (2.8%)	73 (1.3%)	0.10
Hospital level						
Medical center (teaching hospital)	6454 (47.8%)	23,883 (51.1%)	− 0.07	1185 (67.3%)	3762 (67.6%)	− 0.01
Regional/district hospital	7060 (52.2%)	22,810 (48.9%)	0.07	577 (32.7%)	1803 (32.4%)	0.01
Type of CABG						
On pump	–	–	–	1447 (82.1%)	4577 (82.2%)	< 0.01
Off pump	–	–	–	315 (17.9%)	988 (17.8%)	< 0.01
Endarterectomy				79 (4.5%)	274 (4.9%)	− 0.02
Concomitant valve surgery	–	–	–	136 (7.7%)	316 (5.7%)	0.08
Valve location and type						
Aortic valve replacement	–	–	–	48 (2.7%)	131 (2.4%)	0.02
Mitral valve repair	–	–	–	30 (1.7%)	80 (1.4%)	0.02
Mitral valve replacement	–	–	–	60 (3.4%)	123 (2.2%)	0.07
Details of coronary stenting						
Type of stent						
BMS	10,076 (74.6%)	34,400 (73.7%)	0.02	–	–	–
DES	3438 (25.4%)	12,293 (26.3%)	− 0.02	–	–	–
Number of stenting	1.03 ± 0.17	1.03 ± 0.16	0.03	–	–	–
Number of intervened/grafted vessels						
1	10,118 (74.9%)	37,482 (80.3%)	− 0.13	134 (7.6%)	298 (5.4%)	0.09
2	2984 (22.1%)	8201 (17.6%)	0.11	339 (19.2%)	911 (16.4%)	0.08
3	412 (3.0%)	1010 (2.2%)	0.06	1289 (73.2%)	4356 (78.3%)	− 0.12
Antiplatelet therapy at discharge						
Aspirin	9675 (71.6%)	38,780 (83.1%)	− 0.28	665 (37.7%)	2597 (46.7%)	− 0.18
Clopidogrel/ticlopidine	11,027 (81.6%)	41,574 (89.0%)	− 0.21	425 (24.1%)	1496 (26.9%)	− 0.06
Antiplatelet therapy						
Single	4089 (30.3%)	8675 (18.6%)	0.27	1686 (95.7%)	5204 (93.5%)	0.10
Dual	9425 (69.7%)	38,018 (81.4%)	− 0.27	76 (4.3%)	361 (6.5%)	− 0.10
Other medications at discharge						
Statin	6357 (47.0%)	26,159 (56.0%)	− 0.18	431 (24.5%)	1447 (26.0%)	− 0.04
Beta-blocker	6909 (51.1%)	26,955 (57.7%)	− 0.13	563 (32.0%)	1934 (34.8%)	− 0.06
ACEI/ARB	7683 (56.9%)	28,702 (61.5%)	− 0.09	528 (30.0%)	1492 (26.8%)	0.07
OAC drugs	203 (1.5%)	792 (1.7%)	− 0.02	59 (3.3%)	251 (4.5%)	− 0.06
OHA drugs	4457 (33.0%)	10,819 (23.2%)	0.22	604 (34.3%)	1557 (28.0%)	0.14
Insulin	1333 (9.9%)	1753 (3.8%)	0.24	283 (16.1%)	437 (7.9%)	0.26
PPI	901 (6.7%)	2233 (4.8%)	0.08	111 (6.3%)	299 (5.4%)	0.04
NSAID	2763 (20.4%)	9253 (19.8%)	0.02	284 (16.1%)	1029 (18.5%)	− 0.06
Follow-up (years)	2.8 ± 2.7	3.4 ± 2.8	− 0.21	3.2 ± 3.4	3.8 ± 3.6	− 0.16

Data were given as frequency (percentage) or mean ± standard deviation

*PCI* percutaneous coronary intervention, *CABG* coronary artery bypass grafting, *STD* standardized difference, *COPD* chronic obstructive pulmonary disease, *GI* gastrointestinal, *ICH* intracranial haemorrhage, *BMS* bare metal stent, *DES* drug-eluting stent, *ACEi* angiotensin-converting enzyme inhibitor, *ARB* angiotensin receptor blocker, *OAC* oral anticoagulants, *OHA* oral hypoglycemic agent, *PPI* proton pump inhibitor

Post hoc subgroup analyses were further completed to examine whether the male–female differences between PCI and CABG persisted in various patient groups. The outcomes of interest include AMI, HHF, and repeat revascularization. Subgroup variables of interest include the presence of diabetes, HF, and CKD. All of the statistical analyses were performed using SAS version 9.4 (SAS Institute, Cary, NC, USA).

## Results

### The inclusion of study patients

We identified a total of 67,534 patients who met the inclusion criteria. 60,207 patients had undergone PCI, of whom 13,514 were female and 46,693 were male; 7327 patients had received CABG: 1762 women and 5565 men (Fig. 1). Prior to matching, women and men who had received PCI were followed for a mean length of 2.8 years (standard deviation [SD] 2.7 years) and 3.4 years (SD 2.8 years), respectively. Women and men who had undergone CABG were followed for a corresponding mean length of 3.2 years (SD 3.4 years) and 3.8 years (SD 3.6 years), respectively (Table 1). After matching, 13,058 women and 13,058 men remained in the PCI group, and 1716 women and 1716 men remained in the CABG group. The matched PCI group was followed for a mean length of 2.9 years (SD 2.7 years), whereas the matched CABG group was followed for a mean length of 3.2 years (SD 3.4 years) (Additional file 1: Table S2).

### Baseline characteristics of the PCI and CABG groups

Baseline characteristics of the entire cohorts before propensity score matching are listed in Table 1. Before matching, women were older and had a higher prevalence of most comorbidities (i.e., diabetes, hypertension, HF, PAD, cancers, autoimmune diseases, prior stroke, CKD, and ESRD), except for COPD, which was more prevalent in men. Higher proportions of women than men had a history of major bleeding and gastrointestinal bleeding. CCI scores were also higher in women in both the PCI (3.4 vs. 2.5, STD 0.47) and CABG (3.8 vs. 3.1, STD 0.32) groups.

Compared to men, greater proportions of women were discharged with a single antiplatelet agent after PCI (30.3% vs. 18.6%, STD 0.27), as opposed to dual antiplatelet (DAPT) regimens (69.7% vs. 81.4%, STD − 0.27). These differences were greatly attenuated in the CABG group, in which most patients were discharged with a single antiplatelet agent, regardless of sex (women 95.7% vs. men 93.5%, STD 0.10). Furthermore, there were proportionally fewer women discharged with statins, beta-blockers, or angiotensin-converting enzyme inhibitors/angiotensin receptor blockers compared to men in both the PCI and CABG groups. Baseline characteristics were well-balanced between sexes after matching in both the PCI or CABG groups, as confirmed by most STD values being < 0.1 (Additional file 1: Table S2).

### In-hospital outcomes of female versus male patients before and after matching

Prior to matching, the incidence of in-hospital death, ischemic stroke, pneumonia, gastrointestinal bleeding, new-onset dialysis, and prolonged ventilation were all significantly higher in women who underwent PCI, compared to men in the PCI group (Additional file 1: Table S3). Notably, in-hospital mortality, new-onset dialysis, and prolonged ventilation were higher in women by approximately twofold. Similar findings were present in the CABG group, except that pneumonia and gastrointestinal bleeding occurred at similar frequencies in women and men. The use of IABP support was similar across sexes in both the PCI and CABG groups, whereas ECMO was utilized more often in men (PCI: 0.7% vs. 1.2%, OR 0.62, 95% CI 0.50–0.77; CABG: 6.1% vs. 7.8%, OR 0.76, 95% CI 0.61–0.95). Women were hospitalized and stayed in the ICU for longer periods and accrued higher medical expenses than men in both groups. The adverse outcomes related to female sex were more prominent among patients who had received PCI, with significant interaction with the type of revascularization regarding in-hospital mortality, pneumonia, gastrointestinal bleeding, new-onset dialysis, and prolonged ventilation. Increases in LOS, length of ICU stay, and medical expenditures in female patients were more significant among those who had undergone CABG.

After propensity score matching, in-hospital mortality was higher in women than in men in the PCI group, albeit at nominal significance (9.8% vs. 9.1%, OR 1.09, 95% CI 1.001–1.19) (Table 2). On the contrary, the incidences of IABP use (11.0% vs. 12.9%, OR 0.83, 95% CI 0.77–0.90), ECMO support (0.7% vs. 1.1%, OR 0.64, 95% CI 0.49–0.83), and pneumonia were significantly lower in women than in men in the PCI group. The lengths of hospital stay and ICU stay were longer in women regardless of the type of revascularization. The medical expenditures for women who had undergone CABG were significantly higher than for men in the CABG group, but were similar between women and men who had received PCI. There were no differences across sexes in other in-hospital outcomes in either the PCI or CABG groups. The relationships between female sex and LOS (*P* for interaction < 0.001), length of ICU stay (*P* for interaction = 0.007), and medical costs (*P* for interaction = 0.001) were significantly impacted by the treatment group, with greater increases in those who had undergone CABG.

**Table 2:** In-hospital outcomes of the women versus men in the propensity score-matched cohorts

Outcome/subgroup	Women	Men	OR/*B* of women (95% CI)	*P* for interaction
In-hospital mortality				0.188
PCI	1277 (9.8%)	1184 (9.1%)	1.09 (1.001–1.19)	
CABG	360 (21.0%)	371 (21.6%)	0.96 (0.82–1.14)	
IABP support				0.070
PCI	1435 (11.0%)	1691 (12.9%)	0.83 (0.77–0.90)	
CABG	660 (38.5%)	677 (39.5%)	0.96 (0.84–1.10)	
ECMO support				0.236
PCI	95 (0.7%)	147 (1.1%)	0.64 (0.49–0.83)	
CABG	105 (6.1%)	129 (7.5%)	0.80 (0.62–1.05)	
New onset ischemic stroke				0.758
PCI	235 (1.8%)	197 (1.5%)	1.20 (0.99–1.45)	
CABG	72 (4.2%)	57 (3.3%)	1.28 (0.90–1.82)	
Pneumonia				0.694
PCI	995 (7.6%)	1118 (8.6%)	0.88 (0.81–0.96)	
CABG	162 (9.4%)	190 (11.1%)	0.84 (0.67–1.05)	
GI bleeding				0.825
PCI	799 (6.1%)	796 (6.1%)	1.00 (0.91–1.11)	
CABG	74 (4.3%)	71 (4.1%)	1.04 (0.75–1.46)	
New onset dialysis				0.223
PCI	784 (6.0%)	740 (5.7%)	1.07 (0.96–1.18)	
CABG	301 (17.5%)	317 (18.5%)	0.94 (0.79–1.12)	
Prolonged ventilation^a^				0.880
PCI	1265 (9.7%)	1242 (9.5%)	1.02 (0.94–1.11)	
CABG	587 (34.2%)	574 (33.4%)	1.03 (0.90–1.19)	
Hospital stays (days)				< 0.001
PCI	10.6 ± 12.1	10.0 ± 11.8	0.69 (0.40, 0.98)	
CABG	28.3 ± 20.1	25.8 ± 18.8	2.5 (1.2, 3.8)	
ICU duration (days)				0.007
PCI	4.9 ± 6.5	4.8 ± 6.4	0.17 (0.02, 0.33)	
CABG	13.0 ± 11.1	12.1 ± 10.8	0.87 (0.14, 1.60)	
Medical expenditure (USD × 10^3^)				0.001
PCI	7.8 ± 5.6	7.8 ± 5.7	0.04 (− 0.10, 0.17)	
CABG	20.1 ± 9.9	19.3 ± 9.7	0.78 (0.12, 1.44)	

Data were given as frequency (percentage%) or mean ± standard deviation

*IABP* intra-aortic balloon pumping, *ECMO* extra-corporeal membrane oxygenation, *USD* US dollars, *OR* odds ratio, *B* regression coefficient, *CI* confidence interval, *ICU* intensive care unit, *USD* US dollar

^a^The dependence on mechanical ventilation for seven days or longer

### Long-term outcomes of female versus male patients before and after matching

Among patients who received PCI, the occurrence of CV death, ischemic stroke, the primary composite outcome, death from any cause, and HHF were significantly higher for women than for men before matching (Additional file 1: Table S4). Major bleeding and gastrointestinal bleeding were also more frequent in women than men in the PCI group. In contrast, female sex was protective against repeat revascularization (27.9% vs. 34.7%, OR 0.76, 95% CI 0.74–0.79). In the CABG group, the incidences of CV death, AMI, the primary composite outcome, all-cause mortality, HHF, and gastrointestinal bleeding were significantly higher among women than men prior to matching. There were no differences in repeat revascularization between sexes. The detrimental effects of female sex on CV death, ischemic stroke, the primary composite outcome, all-cause death, HHF, and gastrointestinal bleeding were more pronounced in the PCI group (all *P* for interaction < 0.05), whereas the increase in AMI among women was more evident in the CABG group. The reduction of revascularization events in women was seen only among those who had received PCI, with significant modification based on the treatment group (*P* for interaction = 0.002).

**Fig. 2:**
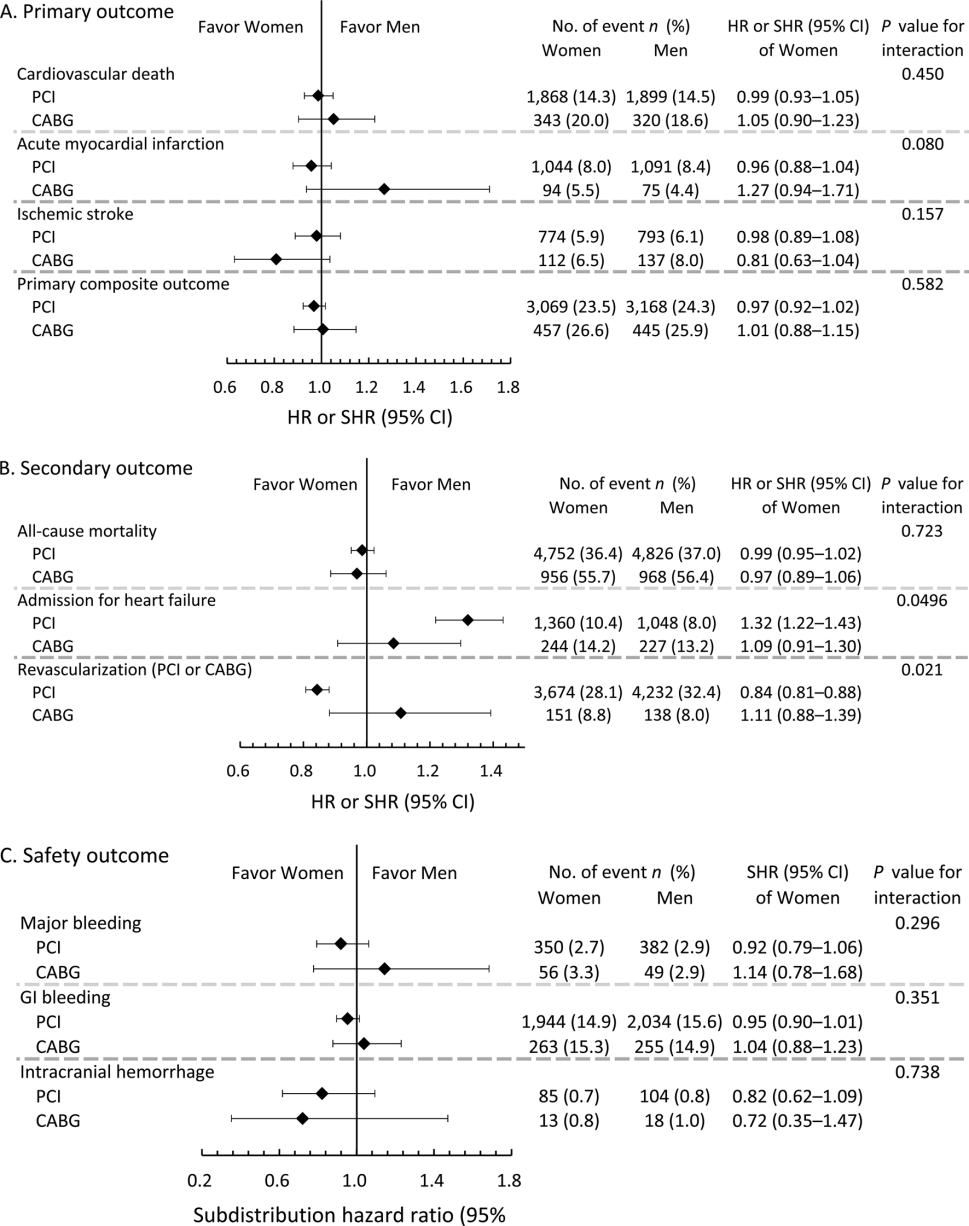
Forest plot comparing the risks of long-term primary outcomes (**A**), secondary outcomes (**B**), and safety outcomes (**C**) between women and men receiving PCI and CABG in the propensity score-matched cohorts

After matching, there were no differences between women and men in the primary outcomes and safety outcomes in either the PCI or CABG groups (Table 3; Fig. 2A, C). In the PCI group, the incidence of HHF was significantly higher in women than in men (10.4% vs. 8.0%, OR 1.32, 95% CI 1.22–1.43), while the occurrence of repeat revascularization was significantly lower (28.1% vs. 32.4%, OR 0.84, 95% CI 0.81–0.88). All-cause mortality and safety outcomes were similar across sexes among those who had received PCI. There were no differences between sexes in terms of secondary and safety outcomes in the CABG group. There was significant interaction between the mode of revascularization and the effects of female sex on the incidences of HHF (*P* for interaction = 0.0496) and repeat revascularization (*P* for interaction = 0.021), with these significant results seen in the PCI group but not the CABG group (Fig. 2B).

**Table 3:** Long-term outcomes of the women versus men in the propensity score-matched cohorts

Outcome/subgroup	Women	Men	HR/SHR of women (95% CI)	*P* for interaction
Primary outcome				
Cardiovascular death				0.450
PCI	1868 (14.3%)	1899 (14.5%)	0.99 (0.93–1.05)	
CABG	343 (20.0%)	320 (18.6%)	1.05 (0.90–1.23)	
Acute myocardial infarction (AMI)				0.080
PCI	1044 (8.0%)	1091 (8.4%)	0.96 (0.88–1.04)	
CABG	94 (5.5%)	75 (4.4%)	1.27 (0.94–1.71)	
Ischemic stroke				0.157
PCI	774 (5.9%)	793 (6.1%)	0.98 (0.89–1.08)	
CABG	112 (6.5%)	137 (8.0%)	0.81 (0.63–1.04)	
Primary composite outcome^a^				0.582
PCI	3069 (23.5%)	3168 (24.3%)	0.97 (0.92–1.02)	
CABG	457 (26.6%)	445 (25.9%)	1.01 (0.88–1.15)	
Secondary outcome				
All-cause mortality				0.723
PCI	4752 (36.4%)	4826 (37.0%)	0.99 (0.95–1.02)	
CABG	956 (55.7%)	968 (56.4%)	0.97 (0.89–1.06)	
Admission for heart failure				0.0496
PCI	1360 (10.4%)	1048 (8.0%)	1.32 (1.22–1.43)	
CABG	244 (14.2%)	227 (13.2%)	1.09 (0.91–1.30)	
Revascularization (PCI or CABG)				0.021
PCI	3674 (28.1%)	4232 (32.4%)	0.84 (0.81–0.88)	
CABG	151 (8.8%)	138 (8.0%)	1.11 (0.88–1.39)	
Safety outcome				
Major bleeding				0.296
PCI	350 (2.7%)	382 (2.9%)	0.92 (0.79–1.06)	
CABG	56 (3.3%)	49 (2.9%)	1.14 (0.78–1.68)	
GI bleeding				0.351
PCI	1944 (14.9%)	2034 (15.6%)	0.95 (0.90–1.01)	
CABG	263 (15.3%)	255 (14.9%)	1.04 (0.88–1.23)	
Intracranial hemorrhage				0.738
PCI	85 (0.7%)	104 (0.8%)	0.82 (0.62–1.09)	
CABG	13 (0.8%)	18 (1.0%)	0.72 (0.35–1.47)	

Data were given as frequency (percentage)

*PCI* percutaneous coronary intervention, *CABG* coronary artery bypass grafting, *HR* hazard ratio, *SHR* subdistribution hazard ratio, *CI* confidence interval, *GI* gastrointestinal

^a^Including cardiovascular death, acute myocardial infarction and ischemic stroke

### Post hoc subgroup analyses of long-term secondary outcomes

The aforementioned primary analysis based on matched cohorts reveals significant interaction effects between sex and the treatment groups on long-term secondary outcomes, including HHF and revascularization. Therefore, the following subgroup analysis was performed on the secondary outcomes, stratified by three clinically relevant comorbidities: diabetes, HF, and CKD. In patients with and without diabetes, the risk of HHF was higher, and the risk of repeat revascularization was lower among women than men in the PCI group (Additional file 1: Table S5). However, these differences in risk between the sexes among those who had received PCI were not significantly different from the results among those who had undergone CABG, except for the risk of HHF, which was more clearly elevated among women in the PCI group than in the CABG group.

**Fig. 3:**
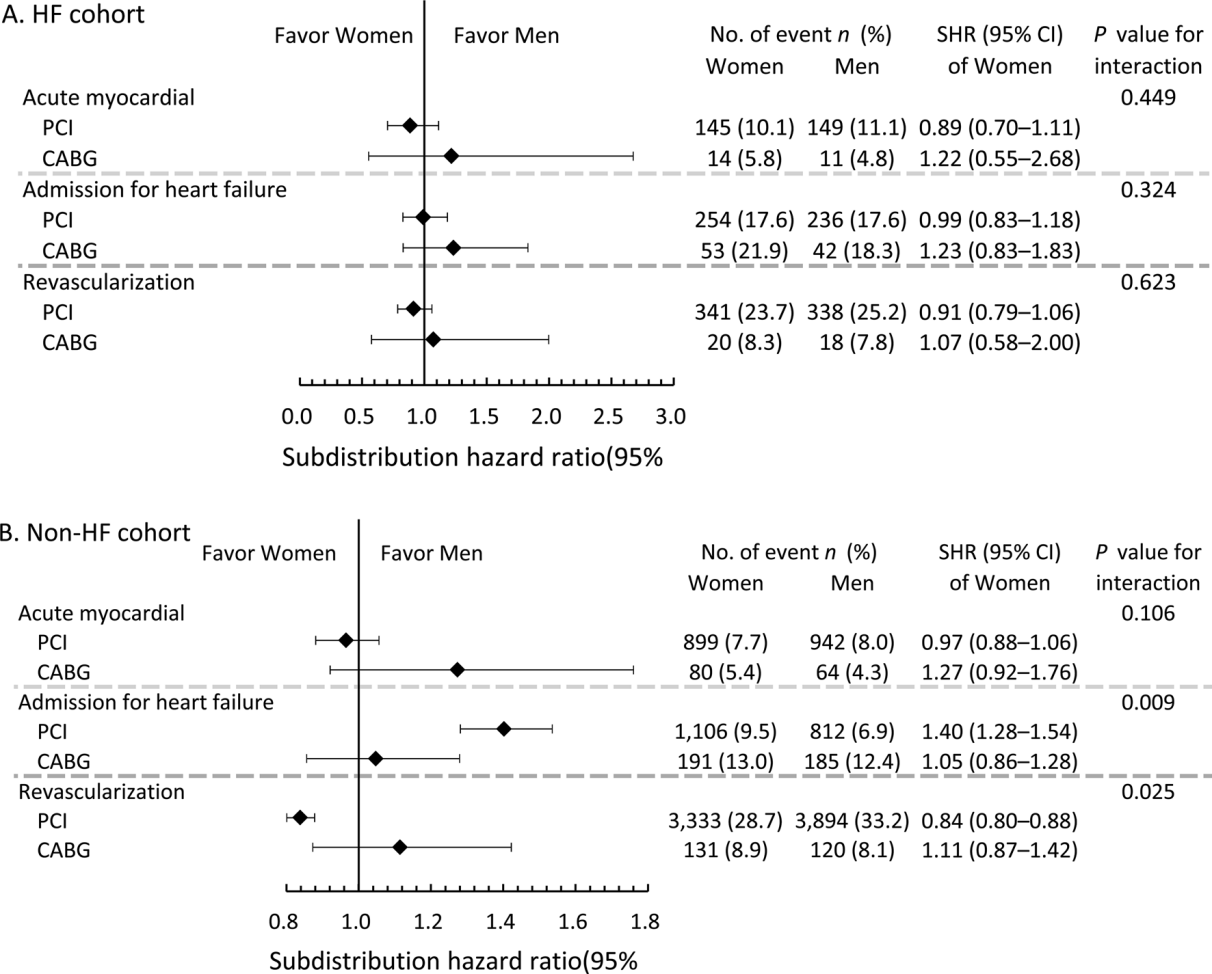
Forest plot comparing the risks of long-term secondary outcomes between women and men receiving PCI and CABG with (**A**) and without (**B**) a history of heart failure hospitalization in the propensity score-matched cohorts

There were no differences between women and men regarding the risks of specified long-term outcomes in either treatment groups for patients with HF at baseline (Additional file 1: Table S6; Fig. 3A). For those with no history of HF, the incidence of HHF was significantly higher—while that of repeat revascularization was significantly lower—among women compared to men in the PCI group (Fig. 3B). These findings were present only in the PCI group, with significant interaction between the effects of female sex and the mode of treatment.

For patients with and without CKD in the PCI group, women were more likely than men to be admitted for heart failure, but less likely than men to require repeat revascularization (Additional file 1: Table S7). The reduced risk of repeat revascularization in women was accentuated in the PCI group compared to the CABG group among those with CKD, whereas the increased risk of HHF for women was more pronounced in the PCI group than in the CABG group among those without CKD.

## Discussion

In this study, we investigated the differences in clinical outcomes of women and men who were first admitted for AMI and who underwent coronary revascularization by PCI or CABG. Prior to matching, enrolled female patients were older, had more comorbid conditions, were more likely to have a significant bleeding history, and were less likely to be discharged with DAPT and optimal medical therapy (OMT) than their male counterparts. In-hospital and long-term outcomes were generally worse among women before matching, particularly for those who had received PCI. After matching, hospital and ICU stay were longer for women in both groups, although this was most pronounced in the CABG group. In the PCI group, IABP and ECMO were more often utilized for men. The incidence of HHF was higher—while that of repeat revascularization was lower—among women, with both effects accentuated among patients who had undergone PCI. Furthermore, this interaction between the influence of female sex and the mode of treatment regarding HHF and repeat revascularization was most prominent in patients without HF at baseline.

The risk of CAD in women increases substantially after menopause, once the protective effects of estrogen have diminished. Hence, women presenting with CAD are often older than men, with multiple comorbidities that accompany aging. Inferior outcomes in women may thus stem from differences in baseline characteristics that should, theoretically, be ameliorated after adjustment for possible confounders or propensity score matching. This was demonstrated in the post hoc analyses of several randomized controlled trials (RCTs), in which the greater hazards of major adverse cardiac events (MACE) or other primary endpoints among women became non-significant after multivariate adjustment [6, 7, 17]. In our study, the incidences of most in-hospital and long-term outcomes were higher in women before matching, but were similar between the two sexes after matching. Thus, it is difficult to delineate the extent to which hormonal differences in females and males contribute to their differing prognoses, or to determine whether or not their underlying medical conditions play much greater roles.

In addition to the effects of comorbidities, the outcomes of CVD among women are also heavily influenced by social, economic, and cultural factors [1]. Women are more likely to receive more conservative treatment than men in similar clinical scenarios. As demonstrated in a retrospective cohort study of patients with AMI, fewer women had undergone revascularization of any type, even after matching for age and type of AMI with men (18). Likewise, in patients with ACS and concomitant high bleeding risk and high ischemic risk, guideline-recommended therapy (including revascularization and DAPT) was given less often to women than men with similar risk profiles [19]. In another report on patients with AMI, a higher proportion of men than women received mechanical circulatory support, despite a higher percentage of women presenting with cardiogenic shock [20]. In our cohort, the use of IABP and ECMO support was significantly higher in men, while in-hospital mortality was nominally higher in women in the PCI group after matching. Although it is difficult to identify the source of these discrepancies, our findings suggest that the treatment of AMI in women may be influenced by sex-specific stereotypes or cultural norms in the real world, and these decisions may be associated with inferior outcomes.

In the present study, women who had undergone PCI were more likely than men to be readmitted for heart failure during long-term follow-up. Sex differences in the susceptibility to heart failure have been extensively described in literature. In a recent report on a large Canadian cohort of patients first admitted for MI, the risks of developing heart failure during admission and long-term follow-up were higher among women after adjustment for underlying conditions, regardless of the type of MI [21]. Similarly, in our cohort, the increase in the risk of heart failure among women remained significant after matching. A higher risk of heart failure among females is observed not only for those with AMI, but also for those with stable CAD. This was demonstrated by Núñez et al. in a study on 5899 individuals with known or suspected CAD who underwent vasodilator stress cardiac magnetic resonance (CMR): women exhibited increased risk of new-onset heart failure at all levels of ischemic or necrosis burden, as shown on CMR[22]. Thus, female sex in itself possibly contributes to the development of heart failure via mechanisms yet to be elucidated, but likely beyond the effects of mere myocardial ischemia.

Interestingly, the higher incidence of HHF in women was not observed in the CABG group. In addition, men who had undergone PCI were more likely to repeat revascularization than women in the same group. These findings suggest fundamental differences in the pathobiology of cardiac ischemia in women and men. As observed in a Swedish registry of 106,881 coronary angiographies performed for ACS, women were more likely to have normal coronary arteries or milder CAD[23]. The prevalence of myocardial infarction with non-obstructive coronary arteries is reportedly higher among women than men for those presenting with ACS[24]. Coronary microvascular dysfunction is hypothesized to be responsible for ischemia with non-obstructive coronary arteries (INOCA) in women. Because patients with less extensive CAD are more likely to receive PCI as opposed to CABG, there is possibly a higher prevalence of INOCA in the PCI group than in the CABG group. Despite seemingly milder disease on angiography, patients with INOCA have an elevated risk for adverse events, particularly the development of heart failure with preserved ejection fraction (HFpEF)[25, 26]. Furthermore, microvascular disease is not treatable by revascularization, and the lower incidence of repeat procedures during follow-up possibly reflects the futility of such treatment. Nonetheless, as mentioned previously, women are likely to receive more conservative treatment, with fewer revascularization procedures. Additional studies are needed to validate these hypotheses.

The results of our subgroup analysis suggest that the discrepancies in the risks of HHF and revascularization in women and men in the PCI group arise primarily from patients without a history of HF. A diagnosis of heart failure, in itself, portends a markedly worsened prognosis. In patients with heart failure, 2- and 5-year mortality have been reported to be 27% and 43%, respectively[27]. Readmission for heart failure within 5 years of HHF reportedly ranges from 40 to 48% across various levels of left ventricular ejection fraction (LVEF) [28]. The hazards accompanying existing HF possibly outweigh the influence of sex on the risks of HHF, while also limiting the possibility for further revascularization procedures, leading to non-significant results in the subgroup of patients with pre-existing HF. On the other hand, as demonstrated by Núñez et al. in their aforementioned study, an increased risk of new-onset heart failure in women was evident only among patients with a baseline LVEF of greater than 55%[22]. In our cohort, data on the LVEF of patients with a diagnosis of HF (or of those admitted for heart failure) are lacking; nonetheless, it is reasonable to believe that those not coded with a diagnosis of HF have a preserved LVEF at baseline. These findings again suggest distinct mechanisms of cardiac ischemia between the sexes, with a possible propensity for microvascular disease and HFpEF in women. Further investigations are necessary to illuminate this subject.

### Perspectives and significance

Woman exhibit greater propensity to heart failure after PCI for AMI compared to men. Women are also less likely to receive mechanical cardiac support in those who underwent PCI despite worse in-hospital survival. Mechanisms underlying cardiac ischemia likely differ between women and men, with greater roles of non-obstructive coronaries and microvascular disease in the female sex. Furthermore, socioeconomic inequalities that influence treatment of female patients are possible. These discrepancies should be considered in the management of CVD in women, and much remains to be explored. Additional investigation is needed to identify sex-specific treatment targets to prevent the development or progression of heart failure in these patients. The choice between PCI and CABG should be guided by evidence and influence from socioeconomic issues should be prevented.

### Limitations

This study has several limitations. First, the study cohort was selected from the NHIRD through ICD-9-CM codes, which provide information on diagnoses, drug use, and procedures. Details such as the severity or risk of the AMI event upon presentation (evaluated by Killip class, TIMI scores or GRACE scores), LVEF in patients coded with HF, or eGFR of patients coded with CKD are lacking. However, we have attempted to account for all possible confounders that may be extracted from the NHIRD data, including the number of arteries intervened, the number of stents placed (in the PCI group), and the details of the surgery (in the CABG group).

Second, only drugs, devices, and services that are fully or partially paid for by the NHI are recorded in the NHIRD. Thus, for the very rare cases where treatments do not fulfill NHI reimbursement criteria, their use would not be identified in our dataset. It should be noted, however, that the NHI reimbursement regulations are set in accordance with global guidelines. Incidents where coinsurance is not possible and a patient must pay completely out-of-pocket are proportionally rare.

Finally, health disparities between the sexes are complex, involving both physiological and socioeconomic differences in women and men. Socioeconomic inferiority may also result in delayed presentation at a later disease stage with more comorbidities. In our study, we seek to describe outcomes for women in general while also exploring physiological responses to the different modes of revascularization in women and men. Although propensity score matching was performed to mitigate differences between the sexes at the time of enrollment, it remains impossible to completely distinguish the physiological and socioeconomic influences of female sex.

## Conclusion

Women presenting with AMI exhibited worse in-hospital and long-term outcomes compared to men, with this disadvantage accentuated among those receiving PCI for revascularization. After matching for underlying characteristics, the incidence of subsequent HHF remained significantly higher in women than in men in the PCI group, while repeat revascularization occurred less often among women. Possible socioeconomic inequalities or distinct pathobiologies of cardiac ischemia between the sexes may underlie these results.

## Supplementary Information

**Additional file 1: Table S1**. ICD-9 CM diagnostic codes for identifying diseases.** Table S2**. Demographics, clinical and surgical characteristics of men versus women in the propensity score matched cohort.** Table S3**. In-hospital outcomes of the women versus men in the cohort before propensity score matching.** Table S4**. Long-term outcomes of the women versus men in the cohort before propensity score matching.** Table S5**. Subgroup analysis (by diabetics) of long-term secondary outcomes of the women versus men in the propensity score matched group.** Table S6**. Subgroup analysis (by history of heart failure hospitalization) of long-term secondary outcomes of the women versus men in the propensity score matched group.** Table S7**. Subgroup analysis (by chronic kidney disease) of long-term secondary outcomes of the women versus men in the propensity score matched group

## Data Availability

The datasets generated and/or analyzed during the current study are available from the corresponding author on reasonable request.

## References

[1] Vogel B, Acevedo M, Appelman Y (2021). The Lancet women and cardiovascular disease Commission: reducing the global burden by 2030. Lancet.

[2] Jin X, Chandramouli C, Allocco B, Gong E, Lam CSP, Yan LL (2020). Women’s participation in cardiovascular clinical trials from 2010 to 2017. Circulation.

[3] Hara H, Takahashi K, van Klaveren D (2020). Sex differences in all-cause mortality in the decade following complex coronary revascularization. J Am Coll Cardiol.

[4] Serruys PW, Cavalcante R, Collet C (2018). Outcomes after coronary stenting or bypass surgery for men and women with unprotected left main disease: the EXCEL trial. JACC Cardiovasc Interv.

[5] Mehran R, Chandrasekhar J, Urban P (2020). Sex-based outcomes in patients with a high bleeding risk after percutaneous coronary intervention and 1-month dual antiplatelet therapy: a secondary analysis of the LEADERS FREE randomized clinical trial. JAMA Cardiol.

[6] Vogel B, Baber U, Cohen DJ (2021). Sex differences among patients with high risk receiving ticagrelor with or without aspirin after percutaneous coronary intervention: a subgroup analysis of the TWILIGHT randomized clinical trial. JAMA Cardiol..

[7] Chichareon P, Modolo R, Kerkmeijer L (2020). Association of sex with outcomes in patients undergoing percutaneous coronary intervention: a subgroup analysis of the GLOBAL LEADERS randomized clinical trial. JAMA Cardiol.

[8] Hsieh C-Y, Su C-C, Shao S-C (2019). Taiwan’s National Health Insurance Research Database: past and future. Clin Epidemiol.

[9] Lin L-Y, Warren-Gash C, Smeeth L, Chen P-C (2018). Data resource profile: the National Health Insurance Research Database (NHIRD). Epidemiol. Health.

[10] Hsing AW, Ioannidis JPA (2015). Nationwide population science: lessons from the Taiwan national health insurance research database. JAMA Intern Med.

[11] Cheng C-L, Lee C-H, Chen P-S, Li Y-H, Lin S-J, Yang Y-HK (2014). Validation of acute myocardial infarction cases in the national health insurance research database in Taiwan. J Epidemiol.

[12] Cheng C-L, Kao Y-HY, Lin S-J, Lee C-H, Lai ML (2011). Validation of the National Health Insurance Research Database with ischemic stroke cases in Taiwan. Pharmacoepidemiol Drug Saf.

[13] Cheng C-L, Chien H-C, Lee C-H, Lin S-J, Yang Y-HK (2015). Validity of in-hospital mortality data among patients with acute myocardial infarction or stroke in National Health Insurance Research Database in Taiwan. Int J Cardiol.

[14] Hsieh C-Y, Chen C-H, Li C-Y, Lai M-L (2015). Validating the diagnosis of acute ischemic stroke in a National Health Insurance claims database. J Formos Med Assoc.

[15] Wu C-S, Lai M-S, Gau SS-F, Wang S-C, Tsai H-J (2014). Concordance between patient self-reports and claims data on clinical diagnoses, medication use, and health system utilization in Taiwan. PLoS ONE.

[16] Sung S-F, Hsieh C-Y, Lin H-J (2016). Validity of a stroke severity index for administrative claims data research: a retrospective cohort study. BMC Health Serv Res.

[17] Rossello X, Ferreira JP, Caimari F (2021). Influence of sex, age and race on coronary and heart failure events in patients with diabetes and post-acute coronary syndrome. Clin Res Cardiol.

[18] de Miguel-Yanes JM, Jiménez-García R, Hernandez-Barrera V (2021). Sex differences in the incidence and outcomes of acute myocardial infarction in Spain, 2016–2018: a matched-pair analysis. J Clin Med.

[19] Mohamed MO, Rashid M, Timmis A (2021). Sex differences in distribution, management and outcomes of combined ischemic-bleeding risk following acute coronary syndrome. Int J Cardiol.

[20] Mahowald MK, Alqahtani F, Alkhouli M (2020). Comparison of outcomes of coronary revascularization for acute myocardial infarction in men versus women. Am J Cardiol.

[21] Ezekowitz JA, Savu A, Welsh RC, McAlister FA, Goodman SG, Kaul P (2020). Is there a sex gap in surviving an acute coronary syndrome or subsequent development of heart failure?. Circulation.

[22] Núñez J, Lorenzo M, Miñana G (2021). Sex differences on new-onset heart failure in patients with known or suspected coronary artery disease. Eur J Prev Cardiol.

[23] Gudnadottir GS, Andersen K, Thrainsdottir IS, James SK, Lagerqvist B, Gudnason T (2017). Gender differences in coronary angiography, subsequent interventions, and outcomes among patients with acute coronary syndromes. Am Heart J.

[24] Trutter L, Bigeh A, Pecci C, Muzaffar M, Gulati M (2020). Diagnostic and management dilemmas in women presenting with acute coronary syndromes. Curr Cardiol Rep.

[25] Bairey Merz CN, Pepine CJ, Walsh MN, Fleg JL (2017). Ischemia and no obstructive coronary artery disease (INOCA): developing evidence-based therapies and research agenda for the next decade. Circulation.

[26] Wei J, Cheng S, Bairey Merz CN (2019). Coronary microvascular dysfunction causing cardiac ischemia in women. JAMA.

[27] Jones NR, Roalfe AK, Adoki I, Hobbs FDR, Taylor CJ (2019). Survival of patients with chronic heart failure in the community: a systematic review and meta-analysis. Eur J Heart Fail.

[28] Shah KS, Xu H, Matsouaka RA (2017). Heart failure with preserved, borderline, and reduced ejection fraction: 5-year outcomes. J Am Coll Cardiol.

