# Supplementary material for: Sex differences following percutaneous coronary intervention or coronary artery bypass surgery for acute myocardial infarction
Source: Biol Sex Differ. 2022 Apr 27;13:18. doi: 10.1186/s13293-022-00427-1 (PMC9044854; doi:10.1186/s13293-022-00427-1)
Supplement: Supplementary file 1 — Additional file 1: Table S1. ICD-9 CM diagnostic codes for identifying diseases. Table S2. Demographics, clinical and surgical characteristics of men versus women in the propensity score matched cohort. Table S3. In-hospital outcomes of the women versus men in the cohort before propensity score matching. Table S4. Long-term outcomes of the women versus men in the cohort before propensity score matching. Table S5. Subgroup analysis (by diabetics) of long-term secondary outcomes of the women versus men in the propensity score matched group. Table S6. Subgroup analysis (by history of heart failure hospitalization) of long-term secondary outcomes of the women versus men in the propensity score matched group. Table S7. Subgroup analysis (by chronic kidney disease) of long-term secondary outcomes of the women versus men in the propensity score matched group [file 13293_2022_427_MOESM1_ESM.docx]

**Supplemental Table 1**. ICD-9 CM diagnostic codes for identifying diseases

| Variable | ICD-9 CM Code |
| --- | --- |
| Myocardial infarction | 410.xx, 412.xx |
| Diabetes mellitus | 250.xx |
| Hypertension | 401.xx–405.xx |
| Dyslipidemia | 272.x |
| Heart failure | 410.xx–414.xx |
| Peripheral arterial disease | 440.0.xx, 440.2x, 440.3x, 440.8x, 440.9x, 443.xx, 444.0x, 444.22, 444.8x, 447.8x, 447.9x |
| Stroke | 430.xx–437.xx |
| Chronic obstructive pulmonary disease | 491.xx, 492.xx, 496.xx |
| Liver cirrhosis | 571.2, 571.5, 571.6 |
| Malignancy | 140.xx–208.xx (Catastrophic illness certificate) |
| Autoimmune disease | 7100, 7101, 7100, 7101, 7102, 7140, 7104, 7103, 4460, 4464, 4465, 4431, 4467, 4461, 1361, 6944, 555.xx, 556, 5560, 5561, 5562, 5563, 5564, 5565, 5566, 5568, 5569, 446.2x (Catastrophic illness certificate) |
| Chronic kidney disease | 580.xx–589.xx, 403.xx–404.xx 016.0x, 095.4x, 236.9x, 250.4x, 274.1x, 442.1x, 447.3x, ,440.1x, 572.4x, 642.1x, 646.2x, 753.1x, 283.11, 403.01, 404.02, 446.21 |
| Dialysis | 585.xx (Catastrophic illness certificate) |
| Major bleeding | 3361, 3636, 37272, 37632, 37742, 37923, 4230, 430, 431, 4320, 4321, 4329, 531, 5312, 5314, 5316, 532, 5322, 5324, 5326, 5307, 533, 5332, 5334, 5336, 534, 5342, 5344, 5346, 5693, 53501, 53511, 53521, 53531, 53541, 53551, 53561, 53571, 53783, 53784, 56202, 56203, 56212, 56213, 56985, 578, 59381, 7191, 72992, 7725, 8520, 8522, 8524, 8530, 86601, 86602, 86611, 86612 |
| Gastrointestinal bleeding | 530.21, 530.7, 530.82, 531.xx–535.xx, 537.83, 537.84, 578.xx |
| Intracranial hemorrhage | 430.xx–432.xx |
| Anemia | 280.xx–284.xx |
| Ischemic stroke | 433.xx–437.xx |
| Pneumonia | 480.xx–486.xx |
| Cardiovascular death | 390.xx–459.xx |
| Acute myocardial infarction | 410.xx |

ICD-9 CM, International Classification of Diseases, Ninth Revision, Clinical Modification.

**Supplemental Table 2.** Demographics, clinical and surgical characteristics of men versus women in the propensity score matched cohort

|  | PCI with stent | | |  | CABG | | |
| --- | --- | --- | --- | --- | --- | --- | --- |
| Variable | Women  (*n* = 13,058) | Men  (*n* = 13,058) | STD |  | Women  (*n* = 1,716) | Men  (*n* = 1,716) | STD |
| Age (years) | 70.9 ± 11.7 | 71.1 ± 11.7 | -0.02 |  | 69.2 ± 9.9 | 69.6 ± 10.2 | -0.04 |
| Comorbid conditions |  |  |  |  |  |  |  |
| Diabetes mellitus | 6,978 (53.4%) | 6,949 (53.2%) | <0.01 |  | 1,167 (68.0%) | 1,159 (67.5%) | 0.01 |
| Hypertension | 10,015 (76.7%) | 10,057 (77.0%) | -0.01 |  | 1,377 (80.2%) | 1,354 (78.9%) | 0.03 |
| Dyslipidemia | 5,709 (43.7%) | 5,596 (42.9%) | 0.02 |  | 657 (38.3%) | 647 (37.7%) | 0.01 |
| Heart failure hospitalization | 1,441 (11.0%) | 1,341 (10.3%) | 0.02 |  | 242 (14.1%) | 230 (13.4%) | 0.02 |
| Peripheral arterial disease | 752 (5.8%) | 751 (5.8%) | <0.01 |  | 132 (7.7%) | 144 (8.4%) | -0.03 |
| Prior stroke hospitalization | 2,396 (18.3%) | 2,365 (18.1%) | 0.01 |  | 342 (19.9%) | 369 (21.5%) | -0.04 |
| COPD | 938 (7.2%) | 1,008 (7.7%) | -0.02 |  | 110 (6.4%) | 117 (6.8%) | -0.02 |
| Liver cirrhosis | 240 (1.8%) | 236 (1.8%) | <0.01 |  | 19 (1.1%) | 22 (1.3%) | -0.02 |
| Malignancy | 741 (5.7%) | 755 (5.8%) | <0.01 |  | 60 (3.5%) | 68 (4.0%) | -0.02 |
| Autoimmune disease | 375 (2.9%) | 366 (2.8%) | <0.01 |  | 41 (2.4%) | 44 (2.6%) | -0.01 |
| Chronic kidney disease | 3,442 (26.4%) | 3,482 (26.7%) | -0.01 |  | 572 (33.3%) | 552 (32.2%) | 0.02 |
| Dialysis | 870 (6.7%) | 854 (6.5%) | <0.01 |  | 109 (6.4%) | 107 (6.2%) | <0.01 |
| Charlson’s Comorbidity Index score | 3.3 ± 2.1 | 3.3 ± 2.1 | -0.01 |  | 3.7 ± 2.0 | 3.7 ± 2.1 | <0.01 |
| Bleeding history |  |  |  |  |  |  |  |
| Major bleeding | 911 (7.0%) | 925 (7.1%) | <0.01 |  | 91 (5.3%) | 92 (5.4%) | <0.01 |
| GI bleeding | 2,574 (19.7%) | 2,599 (19.9%) | <0.01 |  | 292 (17.0%) | 278 (16.2%) | 0.02 |
| ICH | 190 (1.5%) | 193 (1.5%) | <0.01 |  | 21 (1.2%) | 23 (1.3%) | -0.01 |
| Anemia | 430 (3.3%) | 399 (3.1%) | 0.01 |  | 37 (2.2%) | 44 (2.6%) | -0.03 |
| Hospital level |  |  |  |  |  |  |  |
| Medical center (teaching hospital) | 6,282 (48.1%) | 6,291 (48.2%) | <0.01 |  | 1,157 (67.4%) | 1,158 (67.5%) | <0.01 |
| Regional / district hospital | 6,776 (51.9%) | 6,767 (51.8%) | <0.01 |  | 559 (32.6%) | 558 (32.5%) | <0.01 |
| Type of CABG |  |  |  |  |  |  |  |
| On pump | - | - | - |  | 1,413 (82.3%) | 1,411 (82.2%) | <0.01 |
| Off pump | - | - | - |  | 303 (17.7%) | 305 (17.8%) | <0.01 |
| Endarterectomy | 19 (0.15%) | 16 (0.12%) | 0.01 |  | 79 (4.6%) | 78 (4.5%) | <0.01 |
| Concomitant valve surgery | - | - | - |  | 125 (7.3%) | 122 (7.1%) | 0.01 |
| Valve location and type |  |  |  |  |  |  |  |
| Aortic valve replacement | - | - | - |  | 46 (2.7%) | 43 (2.5%) | 0.01 |
| Mitral valve repair | - | - | - |  | 28 (1.6%) | 31 (1.8%) | -0.01 |
| Mitral valve replacement | - | - | - |  | 53 (3.1%) | 51 (3.0%) | 0.01 |
| Details of coronary stenting |  |  |  |  |  |  |  |
| Type of stent |  |  |  |  |  |  |  |
| BMS | 9,726 (74.5%) | 9,713 (74.4%) | <0.01 |  | - | - | - |
| DES | 3,332 (25.5%) | 3,345 (25.6%) | <0.01 |  | - | - | - |
| Number of stenting | 1.03 ± 0.17 | 1.03 ± 0.17 | <0.01 |  | - | - | - |
| Number of intervened/grafted vessels |  |  |  |  |  |  |  |
| 1 | 9,809 (75.1%) | 9,792 (75.0%) | <0.01 |  | 127 (7.4%) | 124 (7.2%) | 0.01 |
| 2 | 2,855 (21.9%) | 2,883 (22.1%) | -0.01 |  | 321 (18.7%) | 332 (19.3%) | -0.02 |
| 3 | 394 (3.0%) | 383 (2.9%) | <0.01 |  | 1,268 (73.9%) | 1,260 (73.4%) | 0.01 |
| Antiplatelet therapy at discharge |  |  |  |  |  |  |  |
| Aspirin | 9,460 (72.4%) | 9,488 (72.7%) | <0.01 |  | 657 (38.3%) | 642 (37.4%) | 0.02 |
| Clopidogrel/Ticodipine | 10,725 (82.1%) | 10,744 (82.3%) | <0.01 |  | 419 (24.4%) | 433 (25.2%) | -0.02 |
| Antiplatelet therapy |  |  |  |  |  |  |  |
| Single | 3,836 (29.4%) | 3,804 (29.1%) | 0.01 |  | 1,640 (95.6%) | 1,645 (95.9%) | -0.01 |
| Dual | 9,222 (70.6%) | 9,254 (70.9%) | -0.01 |  | 76 (4.4%) | 71 (4.1%) | 0.01 |
| Other medications at discharge |  |  |  |  |  |  |  |
| Statin | 6,194 (47.4%) | 6,134 (47.0%) | 0.01 |  | 423 (24.7%) | 413 (24.1%) | 0.01 |
| Beta-blocker | 6,725 (51.5%) | 6,663 (51.0%) | 0.01 |  | 555 (32.3%) | 527 (30.7%) | 0.04 |
| ACEI/ARB | 7,486 (57.3%) | 7,431 (56.9%) | 0.01 |  | 514 (30.0%) | 503 (29.3%) | 0.01 |
| OAC drugs | 198 (1.5%) | 205 (1.6%) | <0.01 |  | 59 (3.4%) | 57 (3.3%) | 0.01 |
| OHA drugs | 4,270 (32.7%) | 4,289 (32.8%) | <0.01 |  | 595 (34.7%) | 590 (34.4%) | 0.01 |
| Insulin | 1,186 (9.1%) | 1,184 (9.1%) | <0.01 |  | 259 (15.1%) | 264 (15.4%) | -0.01 |
| PPI | 857 (6.6%) | 868 (6.6%) | <0.01 |  | 110 (6.4%) | 99 (5.8%) | 0.03 |
| NSAID | 2,670 (20.4%) | 2,658 (20.4%) | <0.01 |  | 280 (16.3%) | 274 (16.0%) | 0.01 |
| Follow-up (years) | 2.9 ± 2.7 | 2.9 ± 2.7 | <0.01 |  | 3.2 ± 3.4 | 3.2 ± 3.3 | 0.02 |

PCI, percutaneous coronary intervention; CABG, coronary artery bypass grafting; STD, standardized difference; COPD, chronic obstructive pulmonary disease; GI, gastrointestinal; ICH, intracranial hemorrhage; BMS, bare-metal stent; DES, drug-eluting stent; ACEi, angiotensin converting enzyme inhibitor; ARB, angiotensin receptor blocker; OAC, oral anticoagulants; OHA, oral hypoglycemic agent; PPI, proton pump inhibitor;

Data were given as frequency (percentage) or mean ± standard deviation.

**Supplemental Table 3.** In-hospital outcomes of the women versus men in the cohort before propensity score matching

| Outcome/ subgroup | Women | Men | OR/ *B* of women (95% CI) | *P* for interaction |
| --- | --- | --- | --- | --- |
| In-hospital mortality |  |  |  | <0.001 |
| PCI | 1,360 (10.1%) | 2,352 (5.0%) | 2.11 (1.97–2.26) |  |
| CABG | 373 (21.2%) | 943 (16.9%) | 1.32 (1.15–1.51) |  |
| IABP support |  |  |  | 0.296 |
| PCI | 1,487 (11.0%) | 4,960 (10.6%) | 1.04 (0.98–1.11) |  |
| CABG | 678 (38.5%) | 2,178 (39.1%) | 0.97 (0.87–1.09) |  |
| ECMO support |  |  |  | 0.193 |
| PCI | 97 (0.7%) | 537 (1.2%) | 0.62 (0.50–0.77) |  |
| CABG | 107 (6.1%) | 435 (7.8%) | 0.76 (0.61–0.95) |  |
| New onset ischemic stroke |  |  |  | 0.156 |
| PCI | 239 (1.8%) | 480 (1.0%) | 1.73 (1.48–2.03) |  |
| CABG | 75 (4.3%) | 174 (3.1%) | 1.38 (1.05–1.82) |  |
| Pneumonia |  |  |  | <0.001 |
| PCI | 1,062 (7.9%) | 2,554 (5.5%) | 1.47 (1.37–1.59) |  |
| CABG | 173 (9.8%) | 550 (9.9%) | 0.99 (0.83–1.19) |  |
| GI bleeding |  |  |  | 0.001 |
| PCI | 833 (6.2%) | 2,060 (4.4%) | 1.42 (1.31–1.55) |  |
| CABG | 76 (4.3%) | 268 (4.8%) | 0.89 (0.69–1.16) |  |
| New onset dialysis |  |  |  | <0.001 |
| PCI | 825 (6.1%) | 1,485 (3.2%) | 1.98 (1.81–2.16) |  |
| CABG | 316 (17.9%) | 858 (15.4%) | 1.20 (1.04–1.38) |  |
| Prolonged ventilation * |  |  |  | <0.001 |
| PCI | 1,355 (10.0%) | 2,692 (5.8%) | 1.82 (1.70–1.95) |  |
| CABG | 613 (34.8%) | 1,617 (29.1%) | 1.30 (1.16–1.46) |  |
| Hospital stays (days) |  |  |  | <0.001 |
| PCI | 10.8 ± 12.3 | 7.8 ± 9.1 | 3.0 (2.8, 3.2) |  |
| CABG | 28.5 ± 20.2 | 24.1 ± 17.6 | 4.4 (3.5, 5.4) |  |
| ICU duration (days) |  |  |  | <0.001 |
| PCI | 5.0 ± 6.6 | 3.7 ± 5.0 | 1.3 (1.2, 1.4) |  |
| CABG | 13.1 ± 11.1 | 11.2 ± 10.1 | 2.0 (1.4, 2.5) |  |
| Medical expenditure (USD×10^3^) |  |  |  | <0.001 |
| PCI | 7.9 ± 5.7 | 6.9 ± 4.6 | 1.0 (0.9, 1.1) |  |
| CABG | 20.2 ± 10.0 | 18.4 ± 9.4 | 1.7 (1.2, 2.3) |  |

IABP, intraaortic balloon pumping; ECMO, extracorporeal membrane oxygenation; USD, US dollars; OR, odds ratio; *B*, regression coefficient; CI, confidence interval; ICU, intensive care unit; USD, US dollar;

* The dependence on mechanical ventilation for seven days or longer;

Data were given as frequency (percentage) or mean ± standard deviation.

**Supplemental Table 4.** Long-term outcomes of the women versus men in the cohort before propensity score matching

| Outcome/ subgroup | Women | Men | HR/ SHR of Women (95% CI) | *P* for interaction |
| --- | --- | --- | --- | --- |
| Primary outcome |  |  |  |  |
| Cardiovascular death |  |  |  | <0.001 |
| PCI | 1,978 (14.6%) | 3,837 (8.2%) | 2.09 (1.98–2.21) |  |
| CABG | 357 (20.3%) | 868 (15.6%) | 1.50 (1.33–1.70) |  |
| Acute myocardial infarction (AMI) |  |  |  | 0.045 |
| PCI | 1,087 (8.0%) | 3,599 (7.7%) | 1.04 (0.98–1.12) |  |
| CABG | 97 (5.5%) | 235 (4.2%) | 1.34 (1.06–1.70) |  |
| Ischemic stroke |  |  |  | <0.001 |
| PCI | 803 (5.9%) | 2,065 (4.4%) | 1.35 (1.25–1.47) |  |
| CABG | 113 (6.4%) | 409 (7.3%) | 0.89 (0.73–1.10) |  |
| Primary composite outcome # |  |  |  | 0.001 |
| PCI | 3,218 (23.8%) | 8,174 (17.5%) | 1.60 (1.54–1.67) |  |
| CABG | 473 (26.8%) | 1,294 (23.3%) | 1.33 (1.20–1.48) |  |
| Secondary outcome |  |  |  |  |
| All-cause mortality |  |  |  | <0.001 |
| PCI | 5,046 (37.3%) | 10,204 (21.9%) | 1.93 (1.86–1.99) |  |
| CABG | 991 (56.2%) | 2,592 (46.6%) | 1.35 (1.25–1.45) |  |
| Admission for heart failure |  |  |  | <0.001 |
| PCI | 1,422 (10.5%) | 2,521 (5.4%) | 2.01 (1.88–2.15) |  |
| CABG | 255 (14.5%) | 607 (10.9%) | 1.39 (1.20–1.60) |  |
| Revascularization (PCI or CABG) |  |  |  | 0.002 |
| PCI | 3,772 (27.9%) | 16,219 (34.7%) | 0.76 (0.74–0.79) |  |
| CABG | 158 (9.0%) | 502 (9.0%) | 1.01 (0.85–1.21) |  |
| Safety outcome |  |  |  |  |
| Major bleeding |  |  |  | 0.504 |
| PCI | 368 (2.7%) | 935 (2.0%) | 1.36 (1.21–1.53) |  |
| CABG | 59 (3.3%) | 157 (2.8%) | 1.22 (0.91–1.64) |  |
| GI bleeding |  |  |  | 0.039 |
| PCI | 2,043 (15.1%) | 5,243 (11.2%) | 1.38 (1.32–1.46) |  |
| CABG | 276 (15.7%) | 762 (13.7%) | 1.19 (1.04–1.36) |  |
| Intracranial hemorrhage |  |  |  | 0.773 |
| PCI | 89 (0.66%) | 319 (0.68%) | 0.96 (0.76–1.21) |  |
| CABG | 14 (0.8%) | 52 (0.9%) | 0.87 (0.49–1.58) |  |

PCI, percutaneous coronary intervention; CABG, coronary artery bypass grafting; HR, hazard ratio; SHR, subdistribution hazard ratio; CI, confidence interval; GI, gastrointestinal;

# including cardiovascular death, acute myocardial infarction and ischemic stroke.

Data were given as frequency (percentage).

**Supplemental Table 5.** Subgroup analysis (by diabetics) of long-term secondary outcomes of the women versus men in the propensity score matched group

| Population/ Outcome/ Subgroup | Women | Men | SHR of Women (95% CI) | *P* for interaction |
| --- | --- | --- | --- | --- |
| Diabetics population |  |  |  |  |
| Acute myocardial infarction (AMI) |  |  |  | 0.247 |
| PCI | 688 (9.9%) | 682 (9.8%) | 1.00 (0.90–1.11) |  |
| CABG | 68 (5.8%) | 55 (4.7%) | 1.24 (0.87–1.77) |  |
| Admission for heart failure |  |  |  | 0.037 |
| PCI | 843 (12.1%) | 637 (9.2%) | 1.33 (1.20–1.48) |  |
| CABG | 174 (14.9%) | 167 (14.4%) | 1.04 (0.85–1.28) |  |
| Revascularization (PCI or CABG) |  |  |  | 0.094 |
| PCI | 2,162 (31.0%) | 2,366 (34.0%) | 0.88 (0.83–0.94) |  |
| CABG | 106 (9.1%) | 95 (8.2%) | 1.12 (0.85–1.47) |  |
| Non-diabetics population |  |  |  |  |
| Acute myocardial infarction (AMI) |  |  |  | 0.168 |
| PCI | 356 (5.9%) | 409 (6.7%) | 0.88 (0.77–1.02) |  |
| CABG | 26 (4.7%) | 20 (3.6%) | 1.33 (0.76–2.36) |  |
| Admission for heart failure |  |  |  | 0.685 |
| PCI | 517 (8.5%) | 411 (6.7%) | 1.30 (1.14–1.48) |  |
| CABG | 70 (12.8%) | 60 (10.8%) | 1.20 (0.86–1.69) |  |
| Revascularization (PCI or CABG) |  |  |  | 0.147 |
| PCI | 1,512 (24.9%) | 1,866 (30.5%) | 0.79 (0.74–0.84) |  |
| CABG | 45 (8.2%) | 43 (7.7%) | 1.08 (0.71–1.63) |  |

PCI, percutaneous coronary intervention; CABG, coronary artery bypass grafting; SHR, subdistribution hazard ratio; CI, confidence interval; GI, gastrointestinal;

Data were given as frequency (percentage).

**Supplemental Table 6.** Subgroup analysis (by history of heart failure hospitalization) of long-term secondary outcomes of the women versus men in the propensity score matched group

| Population/ Outcome/ Subgroup | Women | Men | SHR of Women (95% CI) | *P* for interaction |
| --- | --- | --- | --- | --- |
| Heart failure |  |  |  |  |
| Acute myocardial infarction (AMI) |  |  |  | 0.449 |
| PCI | 145 (10.1%) | 149 (11.1%) | 0.89 (0.70–1.11) |  |
| CABG | 14 (5.8%) | 11 (4.8%) | 1.22 (0.55–2.68) |  |
| Admission for heart failure |  |  |  | 0.324 |
| PCI | 254 (17.6%) | 236 (17.6%) | 0.99 (0.83–1.18) |  |
| CABG | 53 (21.9%) | 42 (18.3%) | 1.23 (0.83–1.83) |  |
| Revascularization (PCI or CABG) |  |  |  | 0.623 |
| PCI | 341 (23.7%) | 338 (25.2%) | 0.91 (0.79–1.06) |  |
| CABG | 20 (8.3%) | 18 (7.8%) | 1.07 (0.58–2.00) |  |
| Non heart failure |  |  |  |  |
| Acute myocardial infarction (AMI) |  |  |  | 0.106 |
| PCI | 899 (7.7%) | 942 (8.0%) | 0.97 (0.88–1.06) |  |
| CABG | 80 (5.4%) | 64 (4.3%) | 1.27 (0.92–1.76) |  |
| Admission for heart failure |  |  |  | 0.009 |
| PCI | 1,106 (9.5%) | 812 (6.9%) | 1.40 (1.28–1.54) |  |
| CABG | 191 (13.0%) | 185 (12.4%) | 1.05 (0.86–1.28) |  |
| Revascularization (PCI or CABG) |  |  |  | 0.025 |
| PCI | 3,333 (28.7%) | 3,894 (33.2%) | 0.84 (0.80–0.88) |  |
| CABG | 131 (8.9%) | 120 (8.1%) | 1.11 (0.87–1.42) |  |

PCI, percutaneous coronary intervention; CABG, coronary artery bypass grafting; SHR, subdistribution hazard ratio; CI, confidence interval; GI, gastrointestinal;

Data were given as frequency (percentage).

**Supplemental Table 7.** Subgroup analysis (by chronic kidney disease) of long-term secondary outcomes of the women versus men in the propensity score matched group

| Population/ Outcome/ Subgroup | Women | Men | SHR of Women (95% CI) | *P* for interaction |
| --- | --- | --- | --- | --- |
| Chronic kidney disease |  |  |  |  |
| Acute myocardial infarction (AMI) |  |  |  | 0.795 |
| PCI | 372 (10.8%) | 360 (10.3%) | 1.05 (0.91–1.22) |  |
| CABG | 32 (5.6%) | 28 (5.1%) | 1.13 (0.68–1.87) |  |
| Admission for heart failure |  |  |  | 0.900 |
| PCI | 468 (13.6%) | 377 (10.8%) | 1.28 (1.12–1.47) |  |
| CABG | 84 (14.7%) | 65 (11.8%) | 1.31 (0.96–1.79) |  |
| Revascularization (PCI or CABG) |  |  |  | 0.048 |
| PCI | 879 (25.5%) | 981 (28.2%) | 0.89 (0.81–0.97) |  |
| CABG | 46 (8.0%) | 33 (6.0%) | 1.39 (0.90–2.16) |  |
| Non chronic kidney disease |  |  |  |  |
| Acute myocardial infarction (AMI) |  |  |  | 0.053 |
| PCI | 672 (7.0%) | 731 (7.6%) | 0.91 (0.82–1.02) |  |
| CABG | 62 (5.4%) | 47 (4.0%) | 1.34 (0.92–1.96) |  |
| Admission for heart failure |  |  |  | 0.013 |
| PCI | 892 (9.3%) | 671 (7.0%) | 1.35 (1.22–1.49) |  |
| CABG | 160 (14.0%) | 162 (13.9%) | 0.99 (0.80–1.24) |  |
| Revascularization (PCI or CABG) |  |  |  | 0.138 |
| PCI | 2,795 (29.1%) | 3,251 (33.9%) | 0.83 (0.79–0.87) |  |
| CABG | 105 (9.2%) | 105 (9.0%) | 1.02 (0.78–1.33) |  |

PCI, percutaneous coronary intervention; CABG, coronary artery bypass grafting; SHR, subdistribution hazard ratio; CI, confidence interval; GI, gastrointestinal;

Data were given as frequency (percentage).
